# Controlling for false positive findings of *trans*-hubs in expression quantitative trait loci mapping

**DOI:** 10.1186/1753-6561-1-s1-s157

**Published:** 2007-12-18

**Authors:** Jie Peng, Pei Wang, Hua Tang

**Affiliations:** 1 Department of Statistics, University of California, Davis, California 95616, USA; 2 Public Health Science, Fred Hutchinson Cancer Research Center, 1100 Fairview Avenue North, Seattle 98109 Washington, USA; 3 Department of Genetics, Stanford University, Stanford, California 94305, USA

## Abstract

In the fast-developing field of expression quantitative traits loci (eQTL) studies, much interest has been concentrated on detecting genomic regions containing transcriptional regulators that influence multiple expression phenotypes (*trans*-hubs). In this paper, we develop statistical methods for eQTL mapping and propose a new procedure for investigating candidate *trans*-hubs. We use data from the Genetic Analysis Workshop 15 to illustrate our methods. After correlations among expressions were accounted for, the previously detected *trans*-hubs are no longer significant. Our results suggest that conclusions regarding regulation hot spots should be treated with great caution.

## Background

The emerging microarray-based gene expression technology enables quantitative geneticists to conduct systematic, genome-wide linkage analysis to detect genomic loci that control gene-expression variations. One of the common features of expression quantitative trait loci (eQTL) studies is the detection of *trans*-hubs, "chromosomal regions that affect the expression of a much larger number of genes than expected by chance" [1]. However, a major concern in *trans*-hub detection is the high false-positive rate due to the complex correlation structure of gene expressions [1]. If a group of genes are highly correlated and a QTL is detected for one of them, then there is a large chance that other expression phenotypes in this group are also mapped to the same QTL, regardless of whether the reason for co-expression is indeed co-regulation at this QTL.

In this paper, we first describe a robust score statistic method designed for three-generation pedigrees for linkage detection. We then introduce a new method for investigating candidate *trans*-hubs. To account for correlations among gene expressions, we treat the expression of additional genes as covariates in the variance-component model of a target gene, and employ sparse regression techniques to remove the covariates' components before testing linkage. The effects of accounting for expression correlations in linkage analysis are illustrated in the Results.

We apply the proposed methods on Genetic Analysis Workshop 15 (GAW15) Problem 1 data [2-4]. This data set consists of 14 three-generation Centre d'Etude du Polymorphisme Humain pedigrees. Genotypes of 2882 autosomal and X-linked SNPs are provided for each individual. Expression levels of ~8500 genes in lymphoblastoid cells were obtained using the Affymetrix Human Focus Arrays [4].

## Methods

### Variance-component model and score statistics

In this section, we derive robust score statistics under the variance-component model [5] to map QTLs. We assumed Hardy-Weinberg equilibrium and linkage equilibrium throughout. The single-locus additive model for a phenotype Y having a mean value *μ *is given by

*Y *= *μ *+ *α*_*m *_+ *α*_*f *_+ *e*,

where *α*_*i *_= *α*_*i*_(*τ*) denotes the additive genetic effect of allele *x *at locus *τ *(the subscript *m *or *f *denotes the parental origin of the allele). We assume that E(*α*_*m*_) = E(*α*_*f*_) = 0, that *α*_*m *_and *α*_*f *_are uncorrelated, and that the residual term *e *is uncorrelated with the explicitly modeled genetic effects. It is straightforward to compute the conditional covariances given the identity-by-decent (IBD) sharing numbers under Eq. (1). For example, for two siblings *i*, *j*, $Cov(Y_{i},Y_{j}|\nu_{ij}(\tau ))=\rho_{s}\sigma_{Y}^{2}+\alpha_{0}(\nu_{ij}(\tau )-1)$, where *ν*_*ij*_(*τ*) is the IBD sharing number between the two siblings at the trait locus *τ*, *ρ*_*s *_is the phenotypic correlation among siblings and $\alpha_{0}=E(\alpha_{m}^{2})=E(\alpha_{f}^{2})$ is the linkage parameter.

For each phenotype *Y*, the null hypothesis of no linkage can be written as *H*_0_: *α*_0 _= 0. The working assumption for the variance-component model is that the conditional distribution of the phenotypes in a pedigree given the pair-wise IBD sharing information at a QTL is multivariate normal. At marker *t *(a putative trait locus), the score statistic for testing *α*_0 _= 0 is then $\ell (t)=\sum_{i=1}^{n}\ell_{i}(t)$, where *n *is the number of pedigrees and

$$\ell_{i}(t)=-\frac{1}{2}tr(\Sigma^{-1}A_{i}(t))+\frac{1}{2}\frac{{\left(Y_{i}-\mu \right)}^{T}}{\sigma_{Y}}\Sigma^{-1}A_{i}(t)\Sigma^{-1}\frac{{\left(Y_{i}-\mu \right)}^{T}}{\sigma_{Y}}.$$

Here, *Y*_*i *_denotes the vector of phenotypes in the *i*^th ^pedigree; *A*_*i*_(*t*) is the IBD sharing matrix for the *i*^th ^pedigree at marker *t*:

$$A_{i}(t)(j,\kappa )=\{\begin{array}{ll} {\hat{\nu }}_{j\kappa }-1 & if\;j\neq \kappa \;and\;\{j,\kappa \}\;is\;a\;sib\;pair,\\ {\hat{\nu }}_{j\kappa }-\frac{1}{2} & if\;\{j,\kappa \}\;is\;a\;grandparent\text{-}grandchild\;pair,\\ 0 & otherwise, \end{array}$$

where ${\hat{\nu }}_{j\kappa }$ is the estimated IBD sharing number between the *j*^th ^and *κ*^th ^member at marker *t*; *μ*, *σ*_Y_, Σ are the phenotypic mean, variance, and correlation matrix, respectively. We consider three different types of phenotypic correlations for the three-generation pedigree: sibship correlation (*ρ*_*s*_); grandparent-grandchild correlation (*ρ*_*g*_); and parent-offspring correlation (*ρ*_*o*_). All of these nuisance parameters are estimated by their corresponding sample estimators. We then standardize the above score by its conditional variance given the phenotypes: $I_{\alpha \alpha }(t)=E_{0}[\ell_{}^{2}(t)|Y_{1},\cdots ,Y_{n}]=\sum_{i=1}^{n}E_{0}[\ell_{i}^{2}(t)|Y_{i}]$. Let *a*_*i*,*t*_(*j*, *κ*) = *A*_*i*_(*t*)(*j*, *κ*), then the calculation of the above quantity involves estimation of *E*[*a*_*i*,*t*_(*j*, *κ*)*a*_*i*,*t*_(*j'*k*'*)]. We identify 11 different types of cross products for two pairs (*j*, *κ*) and (*j'*k*'*) which have a nonzero expectation (e.g., (*j*, *κ*) and (*j'k'*) are two sib pairs with one sibling in common). We pool the same type of cross products across all pedigrees and estimate the above expectation by sample mean.

We then define the robust score statistic at marker t as $Z(t)=\ell (t)/\sqrt{I_{\alpha \alpha }(t)}$, which is asymptotically normally distributed with mean zero and variance one, no matter what the actual phenotypic distribution is. Because we do not know the location of the QTL *τ*, we scan the whole genome with the test statistic: *Z*_max _= max_*t*_*Z*(*t*), where the maximum is taken over all marker loci *t *throughout the genome.

### Investigation of *trans*-hubs

When linkage exists between a genome region and an expression phenotype, the regulation can be "indirect" and act through one or more intermediate genes (that is, this region regulates some intermediate genes and their expression in turn regulate the phenotype). Such indirect regulations are usually less interesting. To detect biologically interesting *trans*-hubs, only direct linkage would be meaningful. In this section, we propose a method to distinguish direct and indirect regulations/linkages.

We will illustrate the idea through a simple example. Consider a system of three components: one candidate QTL (*X*) and two expression phenotypes: *Y*_1_, *Y*_2_. It can be shown that if both *Y*_1 _and *Y*_2 _are linked to *X *and if the linkage strength (defined as the proportion of variation explained by *X*) of *Y*_2 _is no greater than that of *Y*_1_, then the system will match to one of the two models: a) *X *regulates *Y*_1 _and *X *regulates *Y*_2 _(connection between *Y*_1 _and *Y*_2 _is allowed); or b) *X *regulates *Y*_1 _and *Y*_1 _regulates *Y*_2_. (Due to limitation of space, detailed models and proofs are omitted.) We need to distinguish these two models in order to decide whether the linkage between *X *and *Y*_2 _is direct (Model (a)) or indirect (Model (b)). This can be revealed through investigating the residual *R*_21 _of the regression model *Y*_2 _~ *Y*_1_: under Model (a), *R*_21 _links to *X*; while under Model (b), *R*_21 _does not link to *X*. On the other hand, if we consider the regression model *Y*_1 _~ *Y*_2_, the residual *R*_12 _will link to X under both models. However, the linkage might be weak. Therefore, in order to avoid performing unnecessary linkage tests on residuals, which decreases the power, we propose to first order the expression phenotypes with respect to their linkage strength at the candidate hub; and then for each expression trait, only those phenotypes with stronger linkage evidence are used as covariates to derive the corresponding residual in the model below. As a result, for any pair of expression traits, there is only one model having the two traits on the opposite sides of the equation.

According to the above discussion, we introduce the variance-component model

*Y*_*i *_= *μ *+ *Y*_-*i*_*β *+ *α*_*m *_+ *α*_*f *_+ *e*,

where a set of expression phenotypes other than *Y*_*i *_are treated as covariates (*Y*_-*i*_). Define *R*_*i *_= *Y*_*i *_- *Y*_-*i*_*β*. Model (2) becomes *R*_*i *_= *μ *+ *α*_m _+ *α*_*f *_+ *e*, for which the score statistics described previously can be applied to test linkage. Thus, the remaining task is to properly derive *R*_*i*_: the residual of the regression model *Y*_*i *_~ {*Y*_-*i*_}. Because of the high dimensionality of the expression phenotypes ({*Y*_-*i*_}), it is crucial to maintain sparsity in the regression models to avoid over-fitting. For this purpose, we apply a sparse regression technique called elastic net [6] to derive *R*_*i*_. Elastic net aims to minimize the loss function *L*(*λ*_1_, *λ*_2_, *β*) = ||*Y *- *Xβ*||_2_^2 ^+ *λ*_2_||*β*||_2_^2 ^+ *λ*_1_||*β*||_1_. The ridge penalty term encourages a grouping effect: strongly correlated predictors tend to be in or out of the model together; the lasso penalty term enables the algorithm to have a more sparse representation and thus serves as a model selection tool [6].

We propose the following procedure for investigating a candidate *trans*-hub region:

1. Order expression phenotypes according to the linkage strength to this region (based on the score statistics *Z *at the hub) from the largest to the smallest.

2. For the *i*^th ^ordered expression *Y*_(*i*)_, perform Elastic net regression *Y*_(*i*) _~ {*Y*_(*j*)_}_*j*<*i *_with *λ*_2 _= 1 and maximum step k_max_. Record the corresponding residue as *R*_(*i*)_.

3. Perform linkage analysis on {*R*_(*i*)_}_*i *_using the robust score statistics.

4. An expression trait is considered to have a direct linkage to the candidate region if both the original expression *Y*_*i *_and residual *R*_*i *_show significant evidence of linkage.

For the GAW15 application, the maximum step for running elastic net is set to be k_max _= 20, which is the mean optimal step chosen using Mallows' *C*_*P *_criterion [7] among 100 randomly picked regression models (in each model, the expression of one randomly chosen gene is regressed on the expressions of all other genes).

### Data analysis

We first performed an empirical normal quantile transformation for each gene's expression to make them marginally normal [8], for the purpose of improving power of linkage detection. We want to point out that the validity of our test statistic is robust to the distributional assumption of the phenotypes because it is standardized by the conditional variance of the score statistic [9,10].

For genotype data, 1197 SNPs were selected from 22 autosomes, such that the inter-marker distance is at least 0.1 cM to avoid linkage disequilibrium. The resulting map has an average inter-marker space of 3.1 cM, mean heterozygosity of 0.42, and mean missing rate of 3.89%. Merlin [11] was used for IBD inference based on the Rutgers sex average linkage map provided by Sung et al. [12]. Linkage tests were performed for the 3554 most variable genes selected by Morley et al. [4] and gender was used as a covariate.

The threshold at each genome-wide significance level was estimated based on 500 permutations. In each permutation cycle, pedigrees of the same size were permuted; within a pedigree, phenotypes were permuted among individuals of the same type (i.e., generation and gender). We also calculated the thresholds according to a Gaussian approximation with skewness correction [10], which results in similar thresholds (Table 1). We observed that the confidence intervals derived from permutation are wider than what would be expected if all tests were independent. This also suggests that the correlations among gene expressions should not be ignored when examining false positives.

**Table 1:** Number of expressions with at least one significant eQTL

	Genome-wide significance level (point-wise significance level)		
	
	0.05 (4.7 × 10^-6^)	0.01 (3.2 × 10^-7^)	0.001 (6.7 × 10^-9^)
Threshold by Gaussian approximation	4.43	4.98	5.68
Number by chance (95% CI)	177.7 (162.2, 193.2)	35.6 (28.5, 42.6)	3.6 (1.3, 5.8)
			
Original data			
Threshold by permutation	4.36	4.95	5.68
Number by permutation (95% CI)	176.0 (139.0, 224.5)	36.0 (24.0, 56.0)	4.0 (0, 9.0)
Observed number	**235**	**66**	**24**
Sibship only	173	40	13
			
Residual data (9p13.3)			
Threshold by permutation	4.45	5.06	5.81
Number by permutation (95% CI)	180.0 (154.0, 207.0)	36.0 (25.0, 48.0)	4.0 (0, 8.0)
Observed number	**225**	**87**	**26**
			
Residual data (14q32)			
Threshold by permutation	4.46	5.07	5.85
Number by permutation (95% CI)	178.0 (151.5, 205.0)	36.0 (24.0, 47.0)	3 (0, 8.0)
Observed number	**212**	**70**	**23**

## Results

The result of eQTL analysis are summarized in Tables 1 and 2. At the genome-wide 0.001 significance level (point-wise *p*-value < 7 × 10^-9^), we identify 24 expression phenotypes with at least one significant eQTL, among which five overlap with the genes reported in Table 1 of Morley et al. [4] (*TM7SF3*, *HSD17B12*, *CHI3L2*, *DSCR2*, and *DDX17*). We also performed genome scans using only sibship data, which, not surprisingly, resulted in a lower power compared to the analysis using whole pedigrees (Table 1).

**Table 2:** Expression phenotypes with the strongest evidence of linkage from genome scans

Point-wise *p*-value	Gene	Gene location	*cis*/*trans*	eQTL location
<10^-15^	LRAP	5q15	*cis*	Chr 5 (99080578)
<10^-15^	HLA-DQB1	6p21.3	*trans*^a^	Chr 6 (37592767)
<10^-15^	CHI3L2	1p13.3	*cis*	Chr 1 (111704864)
<10^-15^	POMZP3	7q11.23	*cis*	Chr 7 (75651464)
<10^-14^	CSTB	21q22.3	*cis*	Chr 21 (44061921)
<10^-14^	TBC1D8	2q11.2	*trans*^a^	Chr 2 (108214542)
<10^-13^	DSCR2	21q22.3	*trans*	Chr 9 (75300235)
<10^-13^	CRYZ	1p31-p22	*trans*^a^	Chr 1 (67949299)
<10^-11^	EGR2	10q21.1	*trans*	Chr 20 (42643248)
<10^-11^	TM7SF3	12q11-q12	*trans*^a^	Chr 12 (39239200)
<10^-11^	DDX17	22q13.1	*cis*	Chr 22 (39410468)

^a^eQTL resides on the same chromosome of the target gene, although being more than 5 Mb away from the target gene.

**Figure 1:**
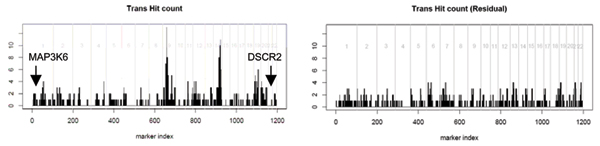
**Distribution of *trans*-hit along the genome**. The x-axis represents the genome order of the 1197 markers. The y-axis represents the number of *trans*-hits in a 5-Mb neighborhood region of each marker. Markers on different chromosomes are separated by vertical gray lines. The left panel is for the original expression data. The right panel is for the residual analysis with respect to 9p13.3. The positions of *DSCR2 *(21q22.3) and *MAP3k6 *(1p36.11), which show strong evidence of *trans*-linkage to 9p13.3 region, are indicated in the left panel.

Following Morley et al. [4], we define *cis*-regulators as the eQTL that map within 5 megabases (Mb) of the target gene and all other eQTL are defined as *trans*-regulators. To illustrate the proposed *trans*-hub investigation method, we examine the *trans*-eQTL events (based on the original expression data) at the genome-wide 0.05 significant level to harvest enough eQTL hits for deriving candidate *trans*-hubs. The numbers of *trans*-hits and *cis*-hits at different significance levels are summarized in Table 3. The number of *trans*-hits dropped dramatically as the significance levels became more stringent, indicating an overall lower confidence of the *tran*s-linkage detection. The genomic locations of the eQTLs detected at the 0.05 significant level suggest two possible hot spots, one at 9p13.3 (30.8 Mb to 35.1 Mb) with 13 *trans*-hits, and another at 14q32 (94.7 Mb to 98.2 Mb) with 11 *trans*-hits (Figure 1, left panel). The latter region, 14q32, has also been recognized as a candidate *trans*-hub by Morley et al. [4]. If regulators for expression phenotypes were independently and randomly distributed along the genome, the probability of the maximum number of hits being at least 13 or 11 are both less than 1 × 10^-6^. However, based on permutation results (in the original data), the chance of observing a *trans*-hub with at least 13 hits is about 11.2%, and the chance of at least 11 hits is as high as 22.4%. These numbers imply that the false detections of *trans*-eQTL are clustered instead of uniformly distributed along the genome. We hypothesize that this is partly due to the expression correlations.

**Table 3:** Number of *cis*-hits and *trans*-hits^a^

		Genome-wide significance level (point-wise significance level)		
		
		0.05 (4.7 × 10^-6^)	0.01 (3.2 × 10^-7^)	0.001 (6.7 × 10^-9^)
*cis*-hits	Original (percentage)	108 (0.72)	74 (0.49)	46 (0.30)
	Residual^b ^(percentage)	182 (1.20)	128 (0.85)	68 (0.45)
				
*trans*-hits	Original (percentage)	1166 (0.028)	296 (0.0070)	96 (0.0023)
	Residual^b ^(percentage)	1057 (0.025)	291 (0.0069)	63 (0.0015)

^a^There are 4,239,027 *trans*-pairs and 15,111 *cis*-pairs in total

^b^Residuals are regarding 9p13.3 (See text for more details.)

To further investigate our hypothesis, we adjusted for the expression correlations as described in the Methods on the *trans*-hub at 9p13.3. The linkage results of residuals are also summarized in Table 1, which show comparable number of eQTL detections as before. At the genome-wide 0.001 significance level, 26 expression phenotypes were identified, of which 9 overlap with the original 24 expression phenotypes mapping to the similar chromosomal regions (*TBC1D8*, *HLA-DPB1*, *CSTB*, *BCKDHA*, *DSCR2*, *POMZP3*, *CHI3L2*, *HSD17B12*, and *TM7SF3*). However, the hub phenomenon becomes much less obvious now: *trans*-eQTL hits were very evenly distributed along the genome (Figure 1, right panel). One explanation is that the overall pair-wise expression correlations in the residual data are much smaller than those in the original data: the median absolute correlation is 0.052 and 0.139, respectively. The maximum number of *trans*-hits of one 5-Mb region at the genome-wide 0.05 significance level is only 4. The number of *trans*-hits at 9p13.3 drops to 3, while among the 500 permutation cycles performed on residual data, none has a maximum number of *trans*-hits smaller than 3. The same analysis is done for the *trans*-hub at 14q32 and the results are similar (Table 1). The number of *trans*-hits at 14q32 drops to 4.

Thus, we conclude that there is not enough evidence to claim either two candidate regions we examined as a *trans*-hub. However, we do find two statistically significant, *trans*-regulated, phenotypes: *DSCR2 *(Down Syndrome Critical Region gene 2) is the most significant (point-wise *p*-value of 10^-12^) gene linked to the 9p13.3 region; *MAP3K6 *also shows strong evidence of linkage to the same region according to both the original phenotype and the residual phenotype. *MAP3K6 *encodes a member of the serine/threonine protein kinase family, and has a point-wise *p*-value of 3 × 10^-7^. The correlation between expressions of *DSCR2 *and *MAP3K6 *is quite small: 0.074. These two *tran*s-regulations may deserve further investigation.

## Discussion

In this paper, we perform linkage analysis for GAW15 data using robust score statistics, which enjoy excellent computational efficiency (20 seconds for computing the score statistics for 3554 expressions on 1197 markers in R on a Thinkpad X40 laptop), and enable us to carry out large-scale permutation studies. Using the original phenotypes, we identify two candidate *trans*-hubs, one at 9p13.3 and the other at 14q32. However, after accounting for the expression correlations in the linkage analysis, both *trans*-hubs disappear. This suggests that conclusions with regard to regulation hot spots should be interpreted with great caution.

Controlling false positives is one of the most important concerns in processing large high dimensional data sets. Without the controlling of false positives, power is not a meaningful quantity. In this paper, we focused on hubs of direct *trans*-regulation, which is conceptually different from the situation where both direct and indirect linkages are sought after. For this purpose, there are two types of false positives: i) the locus and the gene are not linked at all, while a linkage is claimed; ii) the locus and the phenotype are indirectly linked, while a linkage is counted as a direct regulation. The proposed method helps to prevent both types of false positives. Protection against the second type of false positives is discussed in the Methods. As to the first type of false positives, due to correlations among expressions, they do not randomly distribute along the genome. The proposed method also acts as a safeguard against detecting false hubs resulting from this source, since the residuals are usually much less correlated.

## Competing interests

The author(s) declare that they have no competing interests.

## References

[B1] Koning D, Haley C (2005). Genetical genomics in humans and model organisms. Trends Genet.

[B2] Cheung V, Conlin L, Weber T, Arcaro M, Jen K, Morley M, Spielman R (2003). Natural variation in human gene expression assessed in lymphoblastoid cells. Nat Genet.

[B3] Cheung V, Spielman R, Ewens K, Weber T, Morley M, Burdick J (2005). Mapping determinants of human gene expression by regional and whole genome association. Nature.

[B4] Morley M, Molony C, Weber T, Devlin J, Ewens K, Spielman R, Cheung V (2004). Genetic analysis of genome-wide variation in human gene expression. Nature.

[B5] Almasy L, Blangero J (1998). Multipoint quantitative-trait linkage analysis in general pedigrees. Am J Hum Genet.

[B6] Zou H, Hastie T (2005). Regularization and variable selection via the elastic net. J Roy Stat Soc B.

[B7] Mallows L (1973). Some comments on *C_*P*_*. Technometrics.

[B8] Wang K, Huang J (2002). A score-statistic approach for the mapping of quantitative-trait loci with sibships of arbitrary size. Am J Hum Genet.

[B9] Peng J, Siegmund D (2006). Mapping quantitative traits under ascertainment. Ann Hum Genet.

[B10] Tang HK, Siegmund D (2001). Mapping quantitative trait loci in oligogenic models. Biostatistic.

[B11] Abecasis G, Cherny S, Cookson W, Cardon L (2002). Merlin-rapid analysis of dense genetic maps using sparse gene flow trees. Nat Genet.

[B12] Sung YJ, Di Y, Fu AQ, Rothstein JH, Sieh W, Tong L, Thomson EA, Wijsman EM (2007). Comparison of multipoint linkage analyses for quantitative traits in the CEPH data: parametric LOD scores, variance components LOD scores, and Bayes factors. BMC Proc.

